# An unusual case of early speciation associated with human activity

**DOI:** 10.1093/nsr/nwz174

**Published:** 2019-11-13

**Authors:** Wen Wang

**Affiliations:** 1 Center for Ecological and Environmental Sciences, Northwestern Polytechnical University, China; 2 Center for Excellence in Animal Evolution and Genetics, Chinese Academy of Sciences, China; 3 Reviewer of NSR

During the past thousands of years, the expansion of human civilization has changed the surface of Earth. Human activities accelerate the extinction of species, which is called the sixth mass extinction [1]. Some geologists even proposed a new era, Anthropocene, to emphasize the impact of human influence. On the other hand, human activities may also mediate speciation events [2]. Qu *et al.* described an interesting potential speciation event associated with human activities [3]. The story began about 3600 years ago when the rise of barley agriculture in the Qinghai-Tibet Plateau (QTP) [4] seems to have attracted an uninvited guest: the Eurasian tree sparrow, a human commensal species.

Qu *et al.* investigated the highland Eurasian tree sparrow from QTP and compared them with lowland populations. Surprisingly, the harsh environment of QTP, including cold temperature and low oxygen level, did not block off these new colonizers. This is not because the sparrows are born to adapt to high altitudes. Rather, evidence of adaptive evolution was found in the highland populations. The muscle fiber area and capillarization of highland sparrows are significantly increased to enhance the ability of oxygen delivery. A total of 20 muscle-related genes were identified to be under divergent selection in highland populations. Some other muscle-related genes, especially the RhoA/ROCK signaling pathway, were found to be differentially expressed between the two populations. The authors did not find a major adaptive locus associated with novel mutation; it is more likely that the gene-frequency shifts across multiple loci have driven these sparrows to be colonized to QTP. Apart from these signals of adaptive evolution, the whole genome-wide difference between the two populations is so small that no significant population structure could be observed. The demographic analysis suggested that this adaptive evolution was completed within around 3000 years, close to the rise of barley agriculture in QTP.

This work provides an interesting candidate case showing the quick adaptability of species. The highland sparrows have initially acquired the ability to survive on the Plateau and thus the chance of evolving into new species by combing standing genetic variation, other than requiring novel mutation, which implies the importance of genetic diversity, which may enable an organism to adapt to another niche. At the same time, this work shows an example of how human activities could create an unexpected new niche, prompting us to think more broadly about the relationship between human activities and the natural environment.

***Conflict of interest statement***. None declared.

## References

[1] Ceballos G , Ehrlich PR, Barnosky AD et al. Sci Adv 2015; 1: e1400253. 10.1126/sciadv.1400253 PMC4640606 26601195

[2] Bull JW , Maron M. Proc Biol Sci 2016; 283: 1833. 10.1098/rspb.2016.0600 PMC4936035 27358365

[3] Qu YH , Chen CH, Xiong Y et al. Natl Sci Rev 2020; 7: 113–27. 10.1093/nsr/nwz138 PMC8289047 34692022

[4] Chen FH , Dong GH, Zhang DJ et al. Science 2015; 347: 248–50. 25593179 10.1126/science.1259172

